# The Role of PDE8 in T Cell Recruitment and Function in Inflammation

**DOI:** 10.3389/fcell.2021.636778

**Published:** 2021-04-16

**Authors:** Paul M. Epstein, Chaitali Basole, Stefan Brocke

**Affiliations:** ^1^Department of Cell Biology, UConn Health, Farmington, CT, United States; ^2^Department of Immunology, UConn Health, Farmington, CT, United States

**Keywords:** cAMP, phosphodiesterase, PDE8, Raf-1 kinase, T cell motility, leukocyte recruitment, inflammation, integrin adhesion

## Abstract

Inhibitors targeting cyclic nucleotide phosphodiesterases (PDEs) expressed in leukocytes have entered clinical practice to treat inflammatory disorders, with three PDE4 inhibitors currently in clinical use as therapeutics for psoriasis, psoriatic arthritis, atopic dermatitis and chronic obstructive pulmonary disease. In contrast, the PDE8 family that is upregulated in pro-inflammatory T cells is a largely unexplored therapeutic target. It was shown that PDE8A plays a major role in controlling T cell and breast cancer cell motility, including adhesion to endothelial cells under physiological shear stress and chemotaxis. This is a unique function of PDE8 not shared by PDE4, another cAMP specific PDE, employed, as noted, as an anti-inflammatory therapeutic. Additionally, a regulatory role was shown for the PDE8A-rapidly accelerated fibrosarcoma (Raf)-1 kinase signaling complex in myelin antigen reactive CD4^+^ effector T cell adhesion and locomotion by a mechanism differing from that of PDE4. The PDE8A-Raf-1 kinase signaling complex affects T cell motility, at least in part, via regulating the LFA-1 integrin mediated adhesion to ICAM-1. The findings that PDE8A and its isoforms are expressed at higher levels in naive and myelin oligodendrocyte glycoprotein (MOG)_35__–__55_ activated effector T (Teff) cells compared to regulatory T (Treg) cells and that PDE8 inhibition specifically affects MOG_35__–__55_ activated Teff cell adhesion, indicates that PDE8A could represent a new beneficial target expressed in pathogenic Teff cells in CNS inflammation. The implications of this work for targeting PDE8 in inflammation will be discussed in this review.

## Introduction

With over 800 members identified in humans, G protein coupled receptors (GPCRs) constitute a large group of signaling molecules expressed on many cells. Together with their other associated signaling molecules, they are the targets for nearly 35% of approved therapeutics (Sriram and Insel, 2018). The majority of therapeutically targeted GPCRs affect the cAMP signaling pathway which is also a major target of potent anti-inflammatory drugs (Schett et al., 2010; Rabe, 2011; Tenor et al., 2011; De Souza et al., 2012; Milara et al., 2012; Schafer, 2012; Wittmann and Helliwell, 2013; Victoni et al., 2014; Dong et al., 2016; Jarnagin et al., 2016; Tom et al., 2016; Zane et al., 2016a, b; Sriram and Insel, 2018; Blokland et al., 2019). cAMP is involved in many physiological functions and a well-established key regulator of chemotaxis and inflammation (Bourne et al., 1974; Amarandi et al., 2016). Binding of extracellular ligands such as chemokines to Gs-coupled GPCRs leads to cAMP synthesis via activation of adenylyl cyclase and conversion of ATP to cAMP (Weis and Kobilka, 2018). Additionally, T cell activation leads to a temporary upregulation of cAMP which is then degraded by cyclic nucleotide phosphodiesterase (PDE) enzymes (Wang et al., 1978). PDEs constitute the sole group of molecules known to hydrolyze cAMP and hence maintain spatial and temporal control over cAMP gradients within a cell (Baillie, 2009). PDEs are grouped into 11 different gene families considering their action on cAMP or cGMP, structural similarity and mode of regulation (Lerner and Epstein, 2006). While PDEs had been considered early as good targets for anti-inflammatory drugs, bringing specific PDE inhibitors into clinical use has faced decades of challenges (Francis et al., 2011), mostly because of off target effects including emesis (Giembycz, 2000; Beavo et al., 2007). The approval of small molecule inhibitors of PDE4 to treat major immunologic conditions demonstrates a tremendous progress over the last 7 years with indications expanding at an almost yearly pace (Maurice et al., 2014; Baillie et al., 2019). Current clinical practice includes several small molecule compounds that target PDE4 for the therapy of lung disease (COPD) (Hatzelmann et al., 2010; Weis and Kobilka, 2018), psoriatic arthritis and plaque psoriasis (Schett et al., 2012) and topically for atopic dermatitis (Callender et al., 2019). Hence PDE inhibitors are proving to be of great clinical benefit in inflammatory disorders (Ahmad et al., 2015). This review will focus on the selective and shared roles of PDE isoforms during the regulation of T cell motility with emphasis on novel insights of PDE8 functions.

## PDE Expression in Immune Cells

As shown in Table 1 [adapted from Lerner and Epstein (2006)], PDEs are encoded by twenty one genes and are by convention organized into 11 gene families based on their key properties. These include overlap in sequence, specificity for cAMP or cGMP as substrate and their mode of regulation. With alternative splicing as well as the existence of multiple transcription initiation sites, at least 100 different forms of PDE have been cloned and many are expressed in a cell and tissue selective manner. Among the 11 PDE gene families, PDE4, PDE7, and PDE8 selectively hydrolyze cAMP as substrate and degrade it into 5′-AMP, PDE5, PDE6, and PDE9 selectively hydrolyze cGMP as substrate, and PDE1, PDE2, PDE3, PDE10, and PDE11 hydrolyze both cAMP and cGMP as substrate. Of note, the affinity of PDE8 for cAMP is greater than that of any of the other PDE gene families (Fisher et al., 1998a; Hayashi et al., 1998; Soderling et al., 1998a; Gamanuma et al., 2003; Bender and Beavo, 2006).

**TABLE 1:** Pde gene families.

Family^1^	Type	Genes	Km cAMP (μM)	Km cGMP (μM)	Commonly Used Inhibitors^2^	References
PDE1	CaM-Dependent	1A 1B 1C	50–100 7–24 1	5 3 1	Vinpocetine (5–25) 8-MM-IBMX (4)	Wang et al., 1990; Yan et al., 1995; Loughney et al., 1996; Sharma and Hickie, 1996; Yu et al., 1997; Zhao et al., 1997; Kakkar et al., 1999; Goraya and Cooper, 2005
PDE2	cGMP-Stimulated	2A	30	10	EHNA (1)	Martins et al., 1982; Manganiello et al., 1990b; Mery et al., 1995; Podzuweit et al., 1995
PDE3	cGMP-Inhibited	3A/B	0.2–0.5	0.02–0.2	Cilostamide (0.005) Milrinone (0.3)	Manganiello et al., 1990a; Komas et al., 1996; Kambayashi et al., 2003
PDE4	cAMP-Specific	4A–D	1–4	–	Rolipram (1) RO 20-1724 (2) Piclamilast (0.001) Roflumilast (0.0002–0.0043) Apremilast (0.07) Crisaborole (0.5)	Epstein et al., 1982; Conti and Swinnen, 1990; Conti et al., 1995; Houslay et al., 1998; Wallace et al., 2005; Akama et al., 2009; Man et al., 2009; Hatzelmann et al., 2010; Schafer et al., 2010
PDE5	cGMP-Specific	5A	–	1–5	Sildenafil (0.003) Zaprinast (0.3) Dipyridamole (0.9)	Francis et al., 1990; Kotera et al., 1998; Loughney et al., 1998; Lin et al., 2000; Wang et al., 2001; Bischoff, 2004
PDE6	Photoreceptor	6A–C	–	20	Zaprinast (0.15) Dipyridamole (0.4)	Gillespie and Beavo, 1989; Gillespie, 1990
PDE7	cAMP-Specific	7A/B	0.03–0.2	–	Dipyridamole (42, 7A; 0.5–9, 7B)	Michaeli et al., 1993; Han et al., 1997; Gardner et al., 2000; Hetman et al., 2000b; Sasaki et al., 2000; Wang et al., 2000
PDE8	cAMP-Specific	8A/B	0.04–0.15	–	Dipyridamole (4–9, 8A; 23–40, 8B) PF-4957325 (0.0007, 8A; <0.0003, 8B, >1.5 all other PDEs)	Fisher et al., 1998a; Hayashi et al., 1998; Soderling et al., 1998a; Gamanuma et al., 2003; Bender and Beavo, 2006; Vang et al., 2010; DeNinno et al., 2011; DeNinno, 2012
PDE9	cGMP-Specific	9A	–	0.07–0.39	Zaprinast (30) SCH 51866 (2)	Fisher et al., 1998b; Soderling et al., 1998b; Wang et al., 2003
PDE10	Dual Substrate	10A	0.05–0.26	3–9	Papaverine (0.03)	Fujishige et al., 1999a, b; Loughney et al., 1999; Soderling et al., 1999
PDE11	Dual Substrate	11A	1–6	0.5–4	Tadalafil (0.07) Zaprinast (11–33) Dipyridamole (0.3–1.8)	Fawcett et al., 2000; Hetman et al., 2000a; Yuasa et al., 2000; Loughney et al., 2005; Weeks et al., 2005

*^1^Shown in this table [adapted from Lerner and Epstein (2006)] are the eleven PDE gene families, the known genes within them, their affinity constants for cAMP and cGMP, and commonly used pharmacological inhibitors for each of the families. The substrate affinity constants listed are approximate and where ranges are given, they represent the range of different Kms reported for these family members in the references cited. ^2^The numbers in parentheses are the approximate reported Kis or IC_50_s for inhibition of that PDE gene family, given in μM. Note: the non-specific methylxanthine inhibitor IBMX inhibits all of the PDE families with the notable exception of the PDE8 and PDE9 gene families, which appear to be resistant to IBMX inhibition.*

### PDE4

**FIGURE 1:**
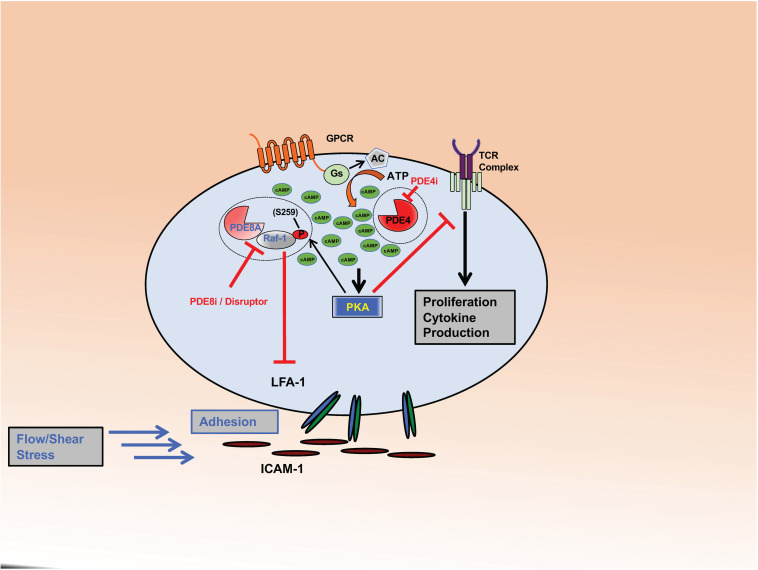
PDE8 regulates Teff cell adhesion. The complex formed between PDE8A and Raf-1 controls adhesion of activated Teff cells through the establishment of tethers between integrin LFA-1 and ICAM-1. PKA becomes activated through the cAMP signaling pathway in response to ligand binding to GPCRs or the Tell receptor. PKA phosphorylates Raf-1 at S259 (P) which inhibits its function to promote adhesion. PDE8A in complex with Raf-1 protects Raf-1 from inhibitory PKA phosphorylation by locally degrading cAMP. PDE8 inhibition or complex disruption leads to an increase of cAMP-dependent PKA activation and Raf-1 phosphorylation at the inhibitory site S259 (P). Action of the PDE8 selective inhibitor PF-04957325 (PDE8i) or disruption of the PDE8A-Raf-1 complex by a signaling disruptor promotes Raf-1 phosphorylation and subsequently inhibits T cell adhesion. This significantly changes Raf-1 activity in T cells. Inhibiting PDE8 targets the tethers formed between LFA-1 and ICAM-1, thereby altering adhesion, spreading and migration of T cells when tested under shear stress conditions. In contrast, PDE4 inhibition (PDE4i) leads to an increase of a pool of cAMP and the activation of PKA signaling localized in signaling complexes that inhibit T cell receptor (TCR) signaling and subsequent T cell activation, cytokine production and proliferation.

Early studies showed PDE4 to be the predominant form of PDE expressed in the cytosolic fraction of human lymphocytes (Epstein and Hachisu, 1984), with PDE4A, 4B, and 4D, but not 4C being expressed (Jiang et al., 1998). PDE4 has long been known to play a key role in regulating T cell activation and functions (Michie et al., 1996; Ekholm et al., 1997; Erdogan and Houslay, 1997; Gantner et al., 1997a, b; Sommer et al., 1997; Barnette et al., 1998; Jin et al., 1998, 2010; Bielekova et al., 2000; Hatzelmann and Schudt, 2001; Kanda and Watanabe, 2001; Arp et al., 2003; Abrahamsen et al., 2004; Asirvatham et al., 2004; Claveau et al., 2004; Jimenez et al., 2004; Bjorgo and Tasken, 2006; Peter et al., 2007; Bjorgo et al., 2010, 2011; Vang et al., 2016). A key mechanism appears to be the modulation of signal transduction through the T cell receptor (TCR). Activation of the TCR leads to cAMP production localized in lipid rafts, activation of cAMP dependent protein kinase A (PKA) and subsequent inhibition of the TCR signal (Abrahamsen et al., 2004; Bjorgo and Tasken, 2006; Wehbi and Tasken, 2016; Figure 1). However, engagement of the co-stimulatory receptor CD28 leads to the recruitment of beta-arrestin and PDE4 to lipid rafts and the decrease of the local cAMP pool and PKA activity. PDE4 inhibitors downregulate the TCR signal by increasing local cAMP concentrations and PKA activity and counteract the CD28-induced recruitment of PDE4. Thus, localized activities of cAMP, PKA, and PDE4 regulate the upstream TCR signal necessary for T cell activation and the subsequent initiation of effector functions.

### PDE3

PDE3 was found to be the predominant form expressed in particulate fractions of human lymphocytes, solely as PDE3B (Tenor et al., 1995; Ekholm et al., 1997; Sheth et al., 1997). In contrast, PDE3B expression is low in regulatory T(reg) cells, and it appears that the low catalytic activity of PDE3B is critical for the regulation of Treg cell-specific gene expression (Gavin et al., 2007). When PDE expression profiles of human T and B lymphocytes were compared, it is of note that in contrast to T cells, which expressed high amounts of PDE3, B cells expressed little or no PDE3 (Gantner et al., 1998).

### PDE7

PDE7, primarily PDE7A, was also found to be expressed in human lymphocytes, to a lesser extent than PDE3 and PDE4, with PDE7A1 primarily cytosolic and PDE7A2 mainly associated with a particulate fraction (Bloom and Beavo, 1996; Giembycz et al., 1996; Bender and Beavo, 2006). Subsequent studies also showed that PDE1B, PDE7A, and PDE8A were all induced following activation of human lymphocytes (Epstein et al., 1980, 1987; Jiang et al., 1998; Glavas et al., 2001; Kanda and Watanabe, 2001). A critical role of the induction of PDE7 for a full T cell activation has been reported (Li et al., 1999; Guo et al., 2009; Szczypka, 2020). Inhibition studies using PDE7 selective inhibitors have yielded divergent results and seem to point to a context and other PDE isoform dependent role of PDE7 in T cell functions and T cell mediated inflammation (Li et al., 1999; Nakata et al., 2002; Yang et al., 2003; Nueda et al., 2006; Goto et al., 2009; Guo et al., 2009; Kadoshima-Yamaoka et al., 2009; Redondo et al., 2012; Gonzalez-Garcia et al., 2013; Xu et al., 2016; Martin-Alvarez et al., 2017; Szczypka, 2020).

### PDE8

The expression of PDE8 in immune cells is listed in Table 2. The function of PDE8 in T cells and cancer cells is discussed in detail below.

**TABLE 2:** PDE8A expression and function in immune cells.

Immune cell subpopulation	PDE gene expression	PDE protein expression	Results	References
CD4^+^ T cells	PDE8A1	PDE8A1	Upregulation of PDE8A1 after polyclonal T cell activation	Glavas et al., 2001
Mitogen-activated splenocytes, anti-CD3 activated CD4^+^ T cells	PDE8A		Induction of PDE8A expression in response to stimulus	Dong et al., 2006; Vang et al., 2010
Antigen exposed naïve and memory CD4^+^ T cells	PDE8A		Changes in temporal expression patterns in response to antigen challenge	Vang et al., 2010
CD4^+^ T cells	PDE8A	PDE8A	Association of PDE8A expression and accumulation of sensitized T cells in draining lymph node of in an animal model of allergic airway disease (AAD)	Vang et al., 2016
CD4^–^ T cells	Low PDE8A	Low PDE8A	Control cell population in AAD	Vang et al., 2016
CD4^+^ T cells	High PDE8A	High PDE8A	Hilar lymph node in AAD	Vang et al., 2016
CD4^+^ effector T(eff) cells	Increased PDE8A expression after polyclonal activation	Increased PDE8A expression after polyclonal activation	PDE8A inhibition by enzymatic inhibitor or a PDE8A- Raf-1 kinase complex disruptor decreases Teff cell adhesion and migration under shear stress conditions	Basole et al., 2017
CD4^+^ regulatory T(reg) cells	Low PDE8A (and all other PDEs expressed in T cells)	Low PDE8A (and all other PDEs expressed in T cells)	High cAMP levels in Treg cells	Bopp et al., 2009; Vang et al., 2010; Vang et al., 2013; Basole et al., 2017
T cells in systemic lupus erythematosus (SLE)	PDE8A1		Upregulation of PDE8A1 transcripts in SLE T cells vs. normal controls	Orlowski et al., 2008
Macrophages	PDE8A		Promotes susceptibility to HIV-1 infection	Booiman et al., 2014

Additionally, very low levels of PDE1, PDE2, and PDE5 activity were also detected in CD4^+^ and CD8^+^ human T cells (Tenor et al., 1995).

### Regulatory T(reg) Cells

As mentioned, PDE3 expression was found to be diminished in Treg cells as compared to CD4^+^ T cells (Gavin et al., 2007). Subsequently it was found that gene expression of *Pde1B*, *Pde2A*, *Pde3B*, *Pde4B*, *Pde5A*, *Pde7A*, and *Pde8A* and protein expression of PDE3B, PDE4B2, and PDE8Awere all reduced in Treg cells as compared to CD4^+^ Teff cells (Bopp et al., 2007, 2009; Vang et al., 2010, 2013; Vaeth et al., 2011; Basole et al., 2017). The low expression levels of PDEs in Treg cells and high level of cAMP have been linked to the mechanism by which Treg cells suppress the function of effector T(eff) cells through the direct cell-to-cell transfer of cAMP (Bopp et al., 2007).

## PDE8A But Not PDE4 Is Controlling Activated T Cell Motility

Inhibition of PDEs regulates T cell proliferation, cytokine production and motility (Sommer et al., 1997; Pette et al., 1999; Bielekova et al., 2000; Vang et al., 2010, 2016; Bjorgo et al., 2011; Mosenden and Tasken, 2011; Wehbi and Tasken, 2016; Basole et al., 2017; Figure 1). Only a small number of PDEs are currently targeted by approved therapeutics, and there is a significant knowledge gap about the specific and potentially therapeutic role of other PDE isoforms. This led to the development of novel PDE inhibitors, including those for therapy of inflammatory conditions. Recently, there have been numerous studies on PDE8A and PDE8B, a family of cAMP specific PDEs (Fisher et al., 1998a; Hayashi et al., 1998; Soderling et al., 1998a; Glavas et al., 2001; Kobayashi et al., 2003; Dong et al., 2006, 2010, 2015; Chen et al., 2009; Vang et al., 2010, 2016; DeNinno et al., 2011; Tsai and Beavo, 2012; Brown et al., 2013; Demirbas et al., 2013; Maurice, 2013; Shimizu-Albergine et al., 2016; Basole et al., 2017; Johnstone et al., 2017; Kelly, 2018). PDE8A and B are expressed widely in human tissue (Wang et al., 2008) with functions identified in testosterone and corticosteroid production (Tsai et al., 2010; Demirbas et al., 2013), myocyte contraction (Patrucco et al., 2010), lymphocyte adhesion and chemotaxis (Dong et al., 2006, 2015; Vang et al., 2010, 2013; Basole et al., 2017), memory and coordination (Tsai et al., 2012), human airway smooth muscle regulation (Johnstone et al., 2017), strong association with immune protection against intracellular pathogens (Blanco et al., 2017), brain disorders associated with inflammation (Chimienti et al., 2019) and T cells in systemic lupus erythematosus (SLE) (Orlowski et al., 2008). In a recent study, single nucleotide polymorphisms in the *PDE8* region were found to be associated with Sjögren’s Syndrome (Taylor et al., 2017). T cell activation induces PDE8A1 (Glavas et al., 2001), a PDE isoform with a cAMP affinity up to 100 times higher than PDE4 isoforms (Fisher et al., 1998a; Hayashi et al., 1998; Soderling et al., 1998a; Gamanuma et al., 2003; Bender and Beavo, 2006). This property of the PDE8 family suggests that they may regulate changes of baseline cAMP gradients around cell signaling complexes. The availability of PDE8 inhibitors and disruptors (Table 3) greatly enhanced the ability to conduct studies on the function of PDE8 *in vitro* and *in vivo*.

**TABLE 3:** Broad and selective PDE8 inhibitors.

Inhibitor, selectivity	References
PF-4957325, PDE8A/B	Vang et al., 2010; DeNinno et al., 2011; DeNinno, 2012
BC8–15, Dual PDE4/8	Demirbas et al., 2013
Dual PDE7/8	Jankowska et al., 2017
Multiple PDE8 inhibiting compounds	Vang et al., 2010; DeNinno et al., 2011; DeNinno, 2012
Dipyridamole, PDE 4–8, 10, and 11	Lerner and Epstein, 2006
Multiple 2-chloroadenine derivatives, PDE8A	Huang et al., 2020
Cell-penetrating peptide agent (PPL-008) inhibiting the PDE8A–C-Raf complex	Blair et al., 2019
Stearylated cell-permeable peptide disrupting the Raf-1–PDE8A Complex based on the Raf-1–docking sequence from PDE8A, encompassing residues R454–T465	Brown et al., 2013

It was shown that PDE8A regulates motility of lymphocytes and breast cancer cells, including adhesion to endothelial cells under physiological shear stress and chemotaxis (Dong et al., 2006, 2015; Vang et al., 2010, 2013; Basole et al., 2017). This seems to be a unique feature of PDE8 that is distinct from PDE4 activity (Vang et al., 2016). The therapeutic potency of biologics and compounds interacting with molecular targets on pathogenic T cells has been shown *in vitro* and *in vivo* (Yednock et al., 1992; Brocke et al., 1999; Steinman, 2005; Healy and Antel, 2016). Current observations demonstrate PDE8 to be one of those targets for blocking Teff cell motility and potentially inflammation (Dong et al., 2006, 2015; Vang et al., 2010, 2013, 2016; Basole et al., 2017). The potential role of PDE8 in models of inflammation and cancer is summarized in Table 4.

**TABLE 4:** PDE8 in models of inflammation and cancer.

PDE8 isoform/assay	Model and Function	References
PDE8A/Expression study	Temporal changes of PDE8A expression in CD4^+^ T cell specific for a cytochrome C peptide/I-E^*k*^ antigen complex transferred into wildtype (non-transgenic) mice, activated with antigen *in vivo*.	Vang et al., 2010
PDE8A/Expression study	CD4^+^ and CD4^–^ T cell populations in a model of ovalbumin-induced allergic airway disease in mice	Vang et al., 2016
PDE8 inhibition study *ex vivo* and *in vitro*	T cells responding to myelin oligodendrocyte glycoprotein (MOG)peptide MOG_35–55_^1^	Vang et al., 2016
PDE8A1/PDE8A2 Expression study	*In vivo* MOG_35–55_ activated CD4^+^CD25^–^ effector and CD4^+^CD25^+^ regulatory cells^1^	Basole et al., 2017
PDE8A and B/Regulating T cell adhesion through inhibitor and peptide disruptor study	*In vivo* MOG_35–55_ activated CD4^+^CD25^–^ effector and CD4^+^CD25^+^ regulatory T cells^1^	Basole et al., 2017
PDE 4–8, 10, and 11/Use of broad PDE inhibitor dipyridamole *in vivo* treatment	Treatment of experimental autoimmune encephalomyelitis^2^	Sloka et al., 2013
PDE8A-C-Raf complex/PDE8A-C-Raf complex disruptor PPL-008	Treatment of human malignant MM415 melanoma cell line *in vitro* and a MM415 melanoma xenograft mouse model *in vivo* with cell-penetrating PDE8A-C-Raf complex disruptor peptide agent (PPL-008) leads to reduced phospho-ERK signaling and growth inhibition	Blair and Baillie, 2019; Blair et al., 2019
PDE8A and B/Expression study	Human breast adenocarcinoma estrogen receptor-positive MCF-7 and T-47D cell lines Human breast adenocarcinoma estrogen receptor-negative MDA-MB-123 MB-231 and MDA-MB-435 cell lines	Dong et al., 2015
PDE8A and B, Expression study	Human breast cancer patient biopsies and tissue arrays	Dong et al., 2015
PDE8A and B, Inhibitor study	Inhibition of MDA-MB-231 breast cancer cell migration and wound healing	Dong et al., 2015
PDE 4–8, 10, and 11/Use of the broad PDE inhibitor dipyridamole *in vivo* treatment	Prevention of triple-negative breast-cancer progression in a mouse model	Virgilio et al., 2012; Spano et al., 2013
PDE 4–8, 10, and 11/Use of the broad PDE inhibitor dipyridamole *in vivo* treatment	Delay of breast cancer lesion onset, tumor progression and suppression of lung metastasis in a mouse model	Wang et al., 2013b

*^1^Myelin oligodendrocyte glycoprotein (MOG) peptide MOG_35–55_ is a myelin autoantigen used to induce experimental autoimmune encephalomyelitis (EAE) in vivo and is recognized by T and B cells in multiple sclerosis (MS). ^2^Experimental autoimmune encephalomyelitis (EAE) is an autoimmune disease model for multiple sclerosis (MS).*

## Differential Roles of PDEs in T Cell Adhesion

Previous studies on the role of PDE8 were done using the prototypic chemokine CXCL12 which is known to induce migration of leukocytes, including murine splenocytes (Cinamon et al., 2001). As measured in a transwell assay system CXCL12 stimulates equally the migration of mouse splenocytes, both unstimulated and stimulated with the mitogen concanavalin A (Con A). Interestingly, when probing the cAMP signaling pathway in CXCL12-induced chemotaxis, differential effects were observed on directed leukocyte migration between exposure of cells to a cell permeable cAMP analog vs. stimulation of adenylyl cyclase or inhibition of PDE. While CXCL12 dependent chemotaxis of both naïve and mitogen activated splenocytes was inhibited by direct exposure to dibutyryl cAMP, activated cells were resistant to indirect cAMP regulation through stimulation of adenylyl cyclase or PDE inhibition (Dong et al., 2006). Activation of adenylyl cyclase through Forskolin (Fsk) or PDE inhibition through the broad PDE enzymatic inhibitor IBMX, significantly reduced CXCL12-induced migration of naive but not activated splenocytes. Additionally, chemotaxis of mitogen-activated splenocytes was not inhibited by PDE3-, PDE4-, and PDE7-selective inhibitors. It is important to note that IBMX is a pan-cAMP specific PDE inhibitor that inhibits all known cAMP PDEs with the exception of PDE8 (Soderling et al., 1998a; Soderling and Beavo, 2000). In contrast, dipyridamole (DP), a broad PDE inhibitor that also targets PDE8 (Soderling et al., 1998a), was found to inhibit migration of naïve and activated splenocytes in response to CXCL12 (Dong et al., 2006). Addition of Fsk in combination with DP increased inhibition of chemotaxis of both naïve and activated splenocytes.

Of note, Rp-cAMPS, an inhibitor of PKA, reversed the DP mediated inhibition of splenocyte migration when exposed to CXCL12. DP is also known to inhibit nucleoside transporters, thereby increasing extracellular adenosine which causes increased levels of cAMP in T cells (Wang et al., 2013a). However, in these assays the action of DP on motility was unaffected by extracellular adenosine deaminase, suggesting that the effect of DP is independent of a possible increase in extracellular adenosine. Expression studies of splenocyte and T cell mRNA demonstrated upregulation of PDE4B2, PDE7A1 and A3, and PDE8A1 in response to T cell activation (Glavas et al., 2001; Dong et al., 2006; Vang et al., 2010). The results indicate that PDE8A1 may have a critical function in regulating cAMP pools that control T cell motility (Dong et al., 2006).

## PDE8A Expression in Effector T (Teff) Cells *in vivo*

PDE8 expression was not only seen in unactivated and polyclonally stimulated splenocytes and CD4^+^ T cells *in vitro*, but also in highly purified CD4^+^ T cell populations *in vivo*. In a widely used TCR transgenic mouse model to investigate highly purified T cell populations *in vivo* in the absence of other leukocytes (Ben-Sasson et al., 2009, 2013), it was shown that antigenic activation resulted in PDE3, 4, and 8 expression in the transgenic T cell subset. Expression levels of the PDE8A gene in activated CD4^+^ T cells were up to fifty percent of those of the PDE3B and PDE4B genes (Vang et al., 2010).

## Selective Inhibition of PDE8 Blocks Splenocyte Adhesion

Integrins including αL or α4 chains have been shown to promote firm adhesion to endothelial cell ligands. cAMP is known to regulate expression and activation of integrin molecules on lymphocytes and other leukocyte populations.

As mentioned, DP was found to inhibit PDE8 and leukocyte migration. Incubation with DP led to a significant reduction of the proportion of αL^*hi*^ and α4^*hi*^ Teff cells.

In contrast, IBMX did not significantly reduce integrin surface expression. Similarly, DP reduced the proportion of activated splenocytes to endothelial cells while IBMX did not have the same effect (Vang et al., 2010). Of note, the potent PDE4 inhibitor piclamilast (PICL) and the PDE3 inhibitor motapizone also did not suppress adhesion. Further, no significant effect of the canonical PDE4 inhibitor rolipram was reported when the interactions between activated T cells and immobilized VCAM-1 or endothelial cells were tested. The specific role of PDE8 action in T cell adhesion could be confirmed using PF-4957325, a selective PDE inhibitor with IC_50_ for PDE8A = 0.0007 μM and IC_50_ for PDE8B < 0.0003 μM, and with IC_50_ for all other PDEs > 1.5 μM (Vang et al., 2010; DeNinno et al., 2011; DeNinno, 2012; Tables 1, 3). As with DP, PF-4957325 significantly suppressed T cell blast adhesion to endothelial cells. Interestingly, when tested in T cell proliferation in response to anti-CD3 stimulation, PICL was significantly more potent at inhibiting T cell proliferation in comparison to PF-4957325 at identical concentrations. These data suggest a specific role for PDE8 in regulating Teff cell adhesion to endothelial cells. Subsequently, it was shown that cAMP signaling controls adhesion in these assays which could account for the effect of PDE8 inhibition.

One of the mechanisms by which cAMP regulates T cell adhesion is through the action of PKA which regulates cell surface expression and affinity of integrin molecules and phosphorylates α4 integrin involved in cellular adhesion (Goldfinger et al., 2003; Lim et al., 2007; Chilcoat et al., 2008). Thus, dibutyryl-cAMP, acting through PKA, inhibits T cell adhesion to endothelial cell ligands. The effect of increased cAMP levels on cell adhesion was not reversed by CXCL12 (Vang et al., 2010).

## Endothelial Cells Also Express PDE8A

Cells of the murine brain endothelium-derived cell line bEnd.3, a polyoma virus middle T oncogene expressing endothelioma cell line (Montesano et al., 1990; Takeuchi and Baichwal, 1995) used for *in vitro* adhesion studies (Vang et al., 2010, 2016; Basole et al., 2017), have been shown to express PDE1, 2, 3, 4, 5, and 7 (Ashikaga et al., 1997; Netherton and Maurice, 2005). Additionally, bEnd.3 cells express PDE8A at levels that are about 25 percent of PDE4B, which is comparable to expression of PDE2A, a functionally critical isoform in endothelial cells (Vang et al., 2010). DP did not induce PDE8A in bEnd.3 or T cells. These results are in contrast to PDE4B which was significantly upregulated in both of these cell populations in response to the increase in intracellular cAMP. These differential responses of PDEs to cAMP signaling further point to different and non-overlapping roles of PDE8 and other PDE isoforms within the same cell populations (Vang et al., 2010).

## PDE8A Gene Expression in CD4^+^ CD25^+^ Regulatory T (Treg) Cells

It is well established that Teff cells and Treg cells express significantly different levels of PDEs. The transcription factor forkhead box P3 (Foxp3) expressed in Treg cells has been shown to selectively repress genes, including *PDE* genes, leading to elevated levels of intracellular cAMP (Bopp et al., 2007, 2009; Vaeth et al., 2011).

Remarkably, the Treg cell subsets show significantly lower expression for *Pde1a*, *Pde1b*, *Pde2a*, *Pde 3b*, *Pde 4b*, *Pde5a*, *Pde7a*, and *Pde8a* genes compared to naive Teff cell subsets. In addition to lower expression of PDE3B and PDE4B2, T reg cells express significantly lower levels of PDE8A in comparison to Teff cells (Vang et al., 2013). Consistent with these findings, it was shown that Foxp3 represses PDE3B, while reduced PDE3B expression through genetic means permits normal Treg cell homeostasis and Treg cell-specific gene expression (Gavin et al., 2007). In contrast, Treg and Teff cells express comparable levels of PDE4B3, PDE4D, and PDE7A (Vang et al., 2013). While the regulation of select PDE isoforms including PDE8 through Foxp3 in Treg cells is well established, the exact role of PDE isoforms regulating Treg cell function remains to be elucidated.

## CD4^+^ T Cells Selectively Express PDE8A in Inflammatory Disease

PDE8A but not PDE8B has been shown to be expressed in T cells (Hayashi et al., 1998, 2002; Dong et al., 2006, 2010).

To test the potential of PDE8 as a target to treat inflammation *in vivo*, the expression of PDE8 was determined in draining lymph nodes of mice that were immunized with ovalbumin (OVA) in an allergic airway disease (AAD) model (Vang et al., 2016).

In this mouse model of AAD, clinical and pathologic resolution occurs with long-term exposure to OVA aerosol. T cells accumulate in hilar and inguinal lymph nodes that drain the exposed lung tissue. The expression of PDE8A protein was determined by Western immunoblot in the CD4^+^ T cell population that was isolated from the lymph nodes at various time points after the induction of AAD. It was found that PDE8A was significantly higher in the CD4^+^ population as compared to the CD4^–^ lymph node populations. Importantly, the increased PDE8A expression was only seen in *in vivo* activated CD4^+^ T cell populations, i.e., after antigen exposure, but not in CD4^+^ T cells from naïve lymph nodes.

Taken together, these results demonstrate that PDE8A expression is higher in the hilar CD4^+^ lymph node cell population than in the hilar CD4^–^ lymph node cell population at various stages of AAD. Additionally, expression levels of the *Pde8a* gene were higher during the acute AAD phase than at the late stage tolerance stage of the disease model (Vang et al., 2016).

## PDE8 and PDE4 Inhibition Show Differential Effects on the *in vitro* Adhesion of T Cells to Endothelial Cells

Studies including a combination of selective and broad PDE inhibitors established overlapping and distinct effects of PDEs in T cell adhesion. Specifically, only the use of PDE inhibitors that target PDE8, including the broad PDE8 inhibitor DP and the highly PDE8-selective inhibitor PF-04957325 (Vang et al., 2010), demonstrated significant inhibition of splenocyte and T cell adhesion. Of note, the PDE inhibitor IBMX, which inhibits all known PDEs that hydrolyze cAMP with the exception of PDE8, shows little inhibitory effect when tested in assays measuring T cell adhesion to endothelial cells. Surprisingly, the potent PDE4 inhibitor PICL showed opposite effects both when used alone or in combination with a PDE8 inhibitor by enhancing adhesion of T cells to endothelial cells. Taken together, these studies show that PDE4 and PDE8 inhibitors exert opposite effects on T cell–endothelial cell interactions in adhesion assays (Vang et al., 2016).

## Opposing Effects of PDE8 and PDE4 Inhibitors on Proliferation of Antigen or Anti-CD3 Stimulated T Cells

In contrast to the observations in adhesion assays, T cell proliferation could be significantly inhibited by the PDE4 inhibitor PICL while the PDE8 inhibitor PF-04957325 showed little effect on proliferation in these studies (Vang et al., 2016). This indicates an action of PDE8 inhibitors on T cell adhesion that is independent of the well-documented inhibition of mitogen-activated protein kinase (MAP) kinase signaling by PDE inhibitors (Giembycz et al., 1996; Essayan et al., 1997; Bjorgo et al., 2011). T cells that were stimulated by specific antigen or anti-CD3 *in vitro* responded significantly lower in proliferation assays when exposed to PICL in comparison to exposure of equal doses of PF-04957325. Importantly, the opposing effects that were seen in adhesion assays were not seen in proliferation assays since combining PICL and PF-04957325 led to a small increase in suppression of proliferation (Vang et al., 2016). These results clearly demonstrate differential actions of PDE4 and PDE8 in regulating key T cell functions.

## Regulation of Vascular Adhesion of Teff Cells vs. Treg Cells Through Inhibition of PDE8 Under Physiologic Shear Stress

It was found that Treg cells form significantly stronger tethers to endothelial cells compared to Teff cells (Maganto-Garcia et al., 2011). It was also established that PDE8A expression in Teff cells differed significantly from that in Treg cells (Vang et al., 2013). Based on these observations adhesion and migration of Teff and Treg cells were investigated for the effects of PDE8 inhibition on these processes (Basole et al., 2017). This question was addressed in a mouse model of autoimmune disease using encephalitogenic Teff cell populations mediating experimental autoimmune encephalomyelitis (EAE). T cells were isolated from draining lymph nodes of mice immunized with a myelin derived peptide, myelin oligodendrocyte glycoprotein (MOG)_35__–__55_, an antigen that is used to induce EAE in susceptible strains of mice (Brocke et al., 1996; Biton et al., 2011). PDE8A1 and PDE8A2 expression at the protein level was compared in the fraction of CD4^+^CD25^–^ Teff cells and CD4^+^CD25^+^ Treg cells, and it was shown that there was significantly higher expression of these PDE8 isoforms in Teff cells than in Treg cells (Vang et al., 2013). However, expression of rapidly accelerated fibrosarcoma (Raf)-1 in Teff cells was not significantly different from that in Treg cells (Basole et al., 2017).

Previous studies demonstrated that Raf-1 profoundly regulates cell motility (Juliano et al., 2004). A study addressing the role of PDE8 in this process used T cells that were derived from *Foxp3gfp.KI* mice (Basole et al., 2017). In these mice, expression of FoxP3, a known regulator of the development of functional Treg cell populations, is tagged with green fluorescent protein (GFP). Using Teff and Treg cell populations from these mice immunized with the myelin derived peptide facilitated separate measurements of inhibitor effects on each cell population under identical conditions. A flow chamber model using physiologic shear stress to measure various categories of tethers of T cell populations to bEnd.3 cells was employed. PDE8 inhibition using PF-04957325 significantly reduced firm tethers of CD4^+^Foxp^+^GFP^–^ Teff cells. In contrast, no significant effect of PDE8 inhibition was observed in adhesion assays of CD4^+^Foxp^+^GFP^+^ Treg cells. It is conceivable that the different sensitivity of Teff and Treg cells to PDE8 inhibition when forming firm adhesive tethers is related to the different expression levels of PDE8A in both cell populations (Basole et al., 2017).

## Adhesion of Teff Cells to Endothelial Cells Is Regulated by a PDE8A-Raf-1 Kinase Signaling Complex

The studies on PDE8 inhibition were followed by mechanistic investigations testing the role of the signaling complex formed by PDE8A and Raf-1 kinase in regulating adhesion of Teff and Treg cells to endothelial cells (Basole et al., 2017). Both molecules assemble in the cytoplasm whereby PDE8A protects RAF-1 kinase from PKA mediated inhibitory phosphorylation (Brown et al., 2013; Maurice, 2013). Vascular adhesion of T cells has been shown to be regulated by members of the Ras family of signaling molecules (Brown et al., 2014). Disruption of the PDE8A-Raf-1 complex by a cell permeable peptide specifically engineered to disrupt this complex (Brown et al., 2013) was tested for its effect on CD4^+^ T cell adhesion and migration under shear stress conditions. Adhesion of CD4^+^GFP^–^Foxp3^–^ Teff cells to vascular endothelial cells was significantly decreased by the disruptor peptide in experiments employing CD4^+^ T cells isolated from the draining lymph nodes of *Foxp3gfp.KI* mice immunized with MOG_35__–__55_, whereas a scrambled control peptide was without effect. Disruption of the PDE8A-Raf-1 complex by the peptide similarly decreased adhesion, spreading and locomotion of Teff cells to isolated ICAM-1 molecules. In contrast, the use of PF-04957325 which targets the enzymatic site of PDE8, did not show comparable effects (Basole et al., 2017). In further comparing the disruptor peptide with the PDE8 enzymatic inhibitor, PF-04957325, it was found that adhesion of CD4^+^ Teff or Treg cells via LFA-1 integrin to ICAM-1 was not markedly altered through the exposure to PF-04957325. In contrast the PDE8A-Raf-1 disruptor peptide significantly reduced cell adherence, spreading and locomotion of CD4^+^GFP^–^Foxp3^–^ Teff cells. The disruptor peptide also reduced transient Treg cell adhesion to ICAM-1, while having no effect on firm tether formation and detachment of these cells. Taken together, this indicates the tether formation between LFA-1 and ICAM-1 on T cells and vascular targets is in part regulated by a signaling complex that is formed between PDE8A and Raf-1. The regulation appears to be LFA-1 integrin specific in that no significant effects were observed when the VLA-4–VCAM-1 interactions were examined using the comparable assay systems and cells (Basole et al., 2017).

## Inhibition of PDE8 Catalytic Activity Significantly Suppresses Extracellular Signal-Regulated Kinase (ERK) Phosphorylation in Activated CD4^+^ T Cells

Inhibition of PDE8 catalytic activity and disruption of the complex of PDE8A-Raf-1 were both investigated for their potential to affect Raf-1 or ERK signaling by analyzing Raf-1 phosphorylation at residue S259 that inhibits its activity (Brown et al., 2013; Maurice, 2013) by PKA and the phosphorylation sites known to activate ERK1/2 in CD4^+^ T cells that were treated with PF-04957325 or disruptor peptide and polyclonally activated (Basole et al., 2017). Inhibitory Raf-1 phosphorylation at S259 and activating ERK1/2 phosphorylation at residues Thr202/Tyr204 were determined by Western immunoblot. Inhibitor treatment did not affect phosphorylation of Raf-1, but a significant decrease of the phosphorylation of ERK1/2 was seen after inhibitor treatment of Teff cells. These observations demonstrate an effect of PDE8 on the ERK1/2 signaling pathway as has been seen with PDE4 in T cells (Baillie and Houslay, 2005). Of note, the abundance of PDE8A increased in response to PDE8 inhibition. Similar results were reported for PDE7A, PDE3B, PDE4B, and PDE4D which were increased when cAMP was elevated in cells (Jiang et al., 1998; Lee et al., 2002; Moon et al., 2002), and this upregulation apparently occurs with PDE8A as well.

## PDE8A-Raf-1 Complex Disruption Within Activated Teff Cells Leads to Raf-1 and ERK1/2 Phosphorylation With Opposing Effects

Treatment of CD4^+^ T cells with the complex disruptor peptide increases phosphorylation of Raf-1 at the inhibitory site at S259 while phosphorylation of ERK1/2 increases at activating sites at Thr202/Tyr204 (Basole et al., 2017). Based on these observations, it is conceivable that the PDE8A-Raf-1 complex regulates motility of T cells through Raf-1 and not through the canonical ERK-MAPK pathway. The exact mechanism underlying these experimental results needs to be elucidated since the reported increase in the phosphorylation of ERK within activated Teff cells can be the result of actions of many effectors interacting in this process (Kortum et al., 2013).

## PDE8A and Breast Cancer Cell Motility

It has been observed that stimulation of cAMP signaling, in many cases through inhibition of PDEs, inhibits migration and motility of some types of cells, including fibroblasts (Fleming et al., 2004), epithelial cells (Lyle et al., 2008), endothelial cells (Netherton and Maurice, 2005), melanoma cells (Dua and Gude, 2008), colon cancer cells (Murata et al., 2000), pancreatic cancer cells (Burdyga et al., 2013; Zimmerman et al., 2015), bladder cancer cells (Ou et al., 2014), cervical cancer cells (Lee et al., 2014), and breast cancer cells (Dong et al., 2015). In the breast cancer study a complete analysis of the expression of PDE genes at the mRNA and protein level in established estrogen receptor positive and negative breast cancer cell lines and in patients’ primary breast cancer biopsies by microarray analysis, qPCR, Western blot analysis, immunofluorescence and immunohistochemistry was performed (Dong et al., 2015). Although a wide range of PDE genes were seen to be expressed in some of these breast cancers by these methods, the PDE8A gene was prominently expressed at the mRNA level in all the breast cancer cells as well as all the breast cancer tissues examined. In addition to its prominent expression in breast cancer cells, expression of PDE8A in the form of an AKAP13-PDE8A fusion transcript has been reported to be highly recurrent in colon cancer cells as well (Nome et al., 2013). Breast cancer cell migration was analyzed both by transwell migration and wound healing assays, and was found to be inhibited by several agonistic cAMP analogs, Fsk, and several PDE inhibitors, in particular DP and the PDE8 selective inhibitor, PF-04957325. Therefore, consistent with our observation of PDE8A being important in the regulation of lymphocyte chemotaxis, it may also be an important regulator, and thus an important target for control of breast cancer cell migration as well.

## Conclusion

Numerous studies demonstrate that T cell adhesion and migration under shear stress conditions are regulated by the enzymatic activity of PDE8 proteins. Additionally, the PDE8A-Raf-1 kinase signaling complex has been identified as a functional site for PDE8A controlling T cell motility (Figure 1). Inasmuch as treatment with the peptide designed to disrupt the PDE8A-Raf-1 complex led to phosphorylation of Raf-1 at an inhibitory site but phosphorylation of ERK1/2 at activating sites, it suggests that PDE8 displacement from the complex exerts a Raf-1 dependent but ERK independent effect on T cell motility. Thus, it is conceivable that the effect of PDE8 inhibition on the formation of tethers and directed migration by T cells is mediated by Raf-1 but not ERK. This model is supported by reports demonstrating regulation of cell motility by Raf-1 and B-Raf through Rho GTPases regulating the actin cytoskeleton and focal adhesions (Ehrenreiter et al., 2005; Klein et al., 2008). The studies on PDE8 identify a novel signaling complex of PDE8-RAF regulating CD4^+^ T cell motility. Upon T cell activation through the TCR, Raf-1 links Ras activation to MAPK signaling (Kortum et al., 2013). Of note, B-Raf most strongly interacts with Ras (Marais et al., 1997; Weber et al., 2000) and activates ERK (Pritchard et al., 1995, 2004; Wojnowski et al., 2000) while cell proliferation and ERK activation are independent of Raf-1. In contrast, Raf-1 is capable of activating Rho and inducing subsequent downstream events in migrating cells without MAPK activity (Ehrenreiter et al., 2005). Thus, MAPKs have been known to act independently in specific signaling pathways. Activation of ERK independent of Ras/Raf-1 can also occur through activation of T cells through the TCR (Kortum et al., 2013). An effect on T cell function that is independent of ERK is also suggested by the finding that PDE8 inhibition does not affect T cell proliferation (Vang et al., 2010, 2016). PDE4 inhibition has been shown to suppress T cell proliferation and ERK1 signaling (Norambuena et al., 2009), but T cell motility is little affected (Lerner and Epstein, 2006; Vang et al., 2016). Collectively, reports over the last few years suggest that PDE8 and the PDE8A-Raf-1 signaling complex selectively regulate the motility of T cells but not T cell proliferation and support the notion that PDE8A exerts its action primarily on Raf-1 kinase and not on MAPK signaling.

## Author Contributions

All authors researched the literature, wrote the manuscript, and reviewed and approved the final version. SB prepared the figure. CB and PE reviewed and approved the final figure.

## Conflict of Interest

The authors declare that the research was conducted in the absence of any commercial or financial relationships that could be construed as a potential conflict of interest.
